# From Observational Biology to Functional and Computable Models of Living Systems

**DOI:** 10.1002/cai2.70090

**Published:** 2026-11-15

**Authors:** Linjia Ji, Haili Qian

**Affiliations:** ^1^ Department of Medical Oncology, National Cancer Center/National Clinical Research Center for Cancer/Cancer Hospital Chinese Academy of Medical Sciences and Peking Union Medical College Beijing China; ^2^ Chinese Academy of Medical Sciences and Peking Union Medical College Beijing China; ^3^ State Key Laboratory of Molecular Oncology, National Cancer Center/National Clinical Research Center for Cancer/Cancer Hospital Chinese Academy of Medical Sciences and Peking Union Medical College Beijing China; ^4^ Beijing Key Laboratory of Innovative Drug Research and Translational Study for Breast Cancer Beijing China

**Keywords:** artificial intelligence, computable biological systems, dynamical systems, life functions

## Abstract

The proposed framework moves from fragmented biological evidence toward testable, computable life functions that describe how biological states evolve over time. A stepwise experimental approach progresses from molecular processes to unicellular and multicellular models, with tumor systems serving as models of pathological state transitions. The graphical image was Created with BioRender.com (Ji L. 2026; https://BioRender.com/zaxq2dd).

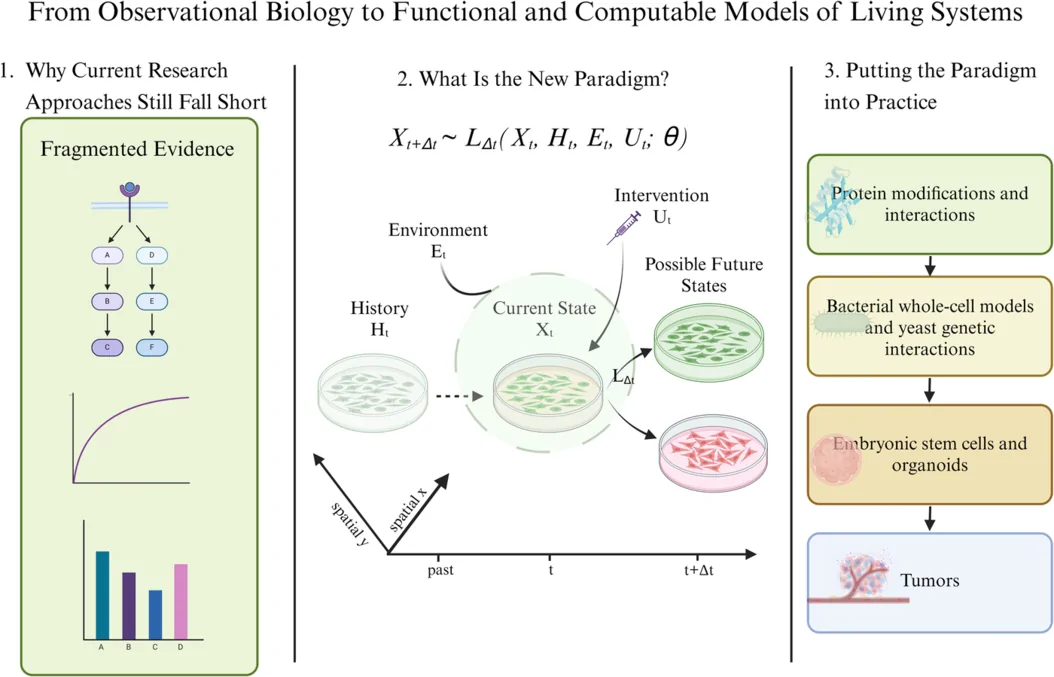

1

Living systems are among the most complex systems known to science. Technological advances in biology have generated vast amounts of data, while artificial intelligence (AI) and other analytical tools have substantially enhanced our ability to analyze these data and extract meaningful insights [1]. However, these advances alone may be insufficient to fundamentally transform how we study and understand living systems. A substantial gap remains between the knowledge generated through biological research and our ability to translate this knowledge into practical applications. Bridging this gap requires a critical reassessment of current research approaches and consideration of whether established conceptual frameworks adequately support the development of functional and computational models of living systems.

## 2 Why Current Research Approaches Still Fall Short

Although causal relationships have been established in many areas of biology, much of biological research still follows a basic sequence of observation, association, and experimental validation. When investigating the relationship between a gene and a biological phenotype, researchers often aim to address gaps in the evidence by identifying signaling pathways or other intermediate mechanisms. However, these approaches do not necessarily enable us to predict how a biological system will respond in different cell types or environments, at different doses, or under combined perturbations. Even the dynamic interactions between two genes can be difficult to characterize quantitatively, let alone the complex interactions among multiple genes. Without a comprehensive understanding of these interactions, an accurate and integrated description of biological processes remains challenging. Experimental studies have identified and functionally validated numerous local, context‐dependent mechanisms. However, these findings remain fragmented and have not yet been systematically integrated into dynamical models with clearly defined state variables, rules governing their evolution over time, and explicit intervention inputs.

Rapid advances in omics technologies, including single‐cell and spatial omics, have enabled more comprehensive characterization of biological systems. These technologies have improved the resolution at which biological states can be described, providing increasingly detailed “snapshots” of living systems. However, the mechanisms governing transitions between these states remain less well understood. Similarly, AI‐based analyses can identify associations between gene expression and phenotypes, but the underlying mechanisms may remain unclear, particularly when the models lack interpretability. Consequently, such analyses may provide limited insight into how biological processes operate, why specific cellular states arise, and how these states evolve over time. Addressing these limitations requires measurable, falsifiable functional relationships among biological components. Establishing such relationships could enable continuous, quantitative, and accurate descriptions of gene–gene and gene–phenotype interactions, improving our ability to understand and predict biological behavior.

## 3 What Is the New Paradigm?

Living systems comprise complex assemblies of structural and functional units organized across multiple scales. Interactions among these units, both within and across molecular, cellular, and tissue levels, provide insights into the principles governing biological systems. Previous studies have established quantitative relationships between molecular signals and cellular responses under specific experimental conditions. For example, following DNA damage, repeated p53 pulses can permit cells to resume proliferation, whereas sustained p53 activation increases the likelihood of cellular senescence [2]. However, these relationships have been characterized in specific cell types and under a limited range of experimental conditions. Further research is needed to integrate such findings into a unified framework capable of describing continuous and discrete transitions between biological states.

To formalize this research objective within a unified, testable framework, we propose drawing on dynamical systems theory and state‐space models to represent living systems as evolving through successive state transitions. We use the term “life function” to describe the mathematical law governing these transitions:

$$X_{t+∆t}\;{\sim \;L}_{∆t}(X_{t},H_{t},E_{t},U_{t};\Theta )$$

Here, $X_{t}$ represents the multiscale state of a living system at time t, encompassing information at the molecular, cellular, tissue, and organismal levels. $H_{t}$ represents the system's history, shaped by development, lineage, aging, and prior stimuli. $E_{t}$ represents environmental and microenvironmental conditions. $U_{t}$ represents external interventions, such as gene editing, drug treatment, nutritional changes, or mechanical stimulation. Θ represents characteristics of the system that are assumed to remain relatively stable over the period studied, including parameters governing the rates and magnitudes of responses, regulatory interactions among components, and feedback mechanisms.

$L_{∆t}$ specifies the state‐transition law over the time interval ∆t and is referred to here as the life function. The symbol ~ indicates that, under specified conditions, the life function defines a probability distribution over possible future states. Thus, rather than representing a single universal equation, the term “life function” refers to a family of state‐transition laws that can be progressively identified, refined, and validated through experimental investigation.

The proposed paradigm emphasizes time‐series measurements and controlled interventions to identify and evaluate candidate life functions. Embryonic development provides a useful context for applying this framework because it involves coordinated processes of cell division, differentiation, and tissue organization [3]. The orderly spatiotemporal progression of cell lineages provides a basis for establishing mathematical relationships that describe these changes and investigating life functions across scales, from microscopic to macroscopic. Organismal aging and epigenetic clocks may also provide insights into these relationships [4].

Development and aging provide a temporal framework for examining physiological and pathological processes across the lifespan. Within this framework, tumorigenesis and other pathological processes can be conceptualized as involving alterations in biological states or the regulatory mechanisms governing transitions between them. This perspective is supported by parallels between several hallmarks of cancer, including sustained proliferation, cellular plasticity, metabolic reprogramming, and immune evasion, and processes involved in normal growth and development, tissue homeostasis, and post‐injury repair [5]. Alterations in regulatory parameters, molecular interactions, and feedback mechanisms may redirect biological systems from physiological trajectories toward pathological states, including those associated with cancer.

The ultimate aim is to move beyond the characterization of biological states toward the identification of testable, dynamic, and computable life functions operating within and across molecular, cellular, and tissue scales. Such functions should provide mechanistic explanations for how biological states emerge and enable predictions of their evolution under different environmental conditions and interventions.

## 4 Putting the Paradigm Into Practice

To develop a comprehensive, dynamic understanding of biological systems, we must first design experiments incorporating controlled interventions and longitudinal measurements to establish functional relationships and investigate interactions among underlying biological processes. These relationships must then be validated under different environmental conditions and combinations of perturbations. First, testable input–output relationships should be established for protein modifications and molecular interactions. Second, bacterial whole‐cell models can be used to integrate diverse biological processes, while yeast genetic interaction studies can help characterize responses to combinatorial perturbations. Third, embryonic stem cell cultures and organoids can be used to investigate biological state transitions and differentiation programs, including how disruptions to these processes may contribute to disease initiation. Fourth, tumors provide heterogeneous, evolving biological systems for examining how alterations in normal regulatory mechanisms and state‐transition dynamics contribute to disease development and progression. Through this stepwise experimental approach, we can progressively develop an integrated understanding of biological systems, extending from molecular processes to unicellular and multicellular models and, ultimately, whole organisms.

Developing a model capable of predicting biological responses does not, in itself, establish a life function. Constructing systems that simulate biological functions requires a fundamental shift in biomedical research toward quantitative models that capture interactions among biological processes. These models must be validated against experimental observations at relevant levels of biological organization. Future studies should focus on developing computational systems that integrate biological functions, allowing individual findings to be evaluated in the context of the broader biological system.

## Author Contributions

**Linjia Ji:** conceptualization, writing – original draft, writing – review and editing. **Haili Qian:** conceptualization, writing – original draft, writing – review and editing.

## Ethics Statement

The authors have nothing to report.

## Consent

The authors have nothing to report.

## Conflicts of Interest

Haili Qian is a member of the *Cancer Innovation* Editorial Board. To minimize bias, he was excluded from all editorial decision‐making related to the acceptance of this article for publication. The co‐author declares no conflicts of interest.

## Data Availability

Data sharing is not applicable to this article because no data sets were generated or analyzed during the study.
